# Erythrocyte Glutathione Shows a Larger Case–Control Difference than Serum Thiol-Disulfide Homeostasis at Emergency Department Presentation with Non-ST-Elevation Myocardial Infarction

**DOI:** 10.3390/antiox15101334

**Published:** 2026-10-16

**Authors:** Faruk Danış, Murat Alışık

**Affiliations:** 1 Department of Emergency Medicine, Faculty of Medicine, Bolu Abant İzzet Baysal University, 14030 Bolu, Türkiye; 2 Department of Medical Biochemistry, Faculty of Medicine, Bolu Abant İzzet Baysal University, 14030 Bolu, Türkiye; muratalisik@ibu.edu.tr

**Keywords:** oxidative stress, thiol-disulfide homeostasis, glutathione, erythrocyte, redox compartmentalization, non-ST-elevation myocardial infarction, emergency department, case–control study

## Abstract

Clinical redox studies usually sample a single compartment. In a single-centre, cross-sectional case–control study, we measured serum thiol-disulfide homeostasis, a bulk extracellular thiol measure, and erythrocyte glutathione, an intracellular measure, in the same blood draw from 82 patients presenting to an emergency department with non-ST-elevation myocardial infarction and 75 controls. Cases and controls differed substantially in sex and in cardiovascular risk profile. All nine redox indices differed between groups in the direction consistent with greater oxidation in patients. The standardized case–control difference was larger for erythrocyte reduced glutathione (Hedges g 1.14) than for serum native thiol (0.56). The post hoc bootstrap ratio of the two was 2.04 (95% confidence interval [CI] 1.31 to 4.08) before adjustment and 1.60 (0.99 to 3.23) after adjustment for age, sex and smoking, and no asymmetry was seen in the small subgroup free of recorded risk factors. In exploratory analyses within patients, serum indices were associated with the Synergy between Percutaneous Coronary Intervention with TAXUS and Cardiac Surgery (SYNTAX) score and reduced glutathione with ejection fraction. Each assay reports its own pool and is not a global measure of oxidative stress. In this sample, the compartment chosen determined the size of the observed case–control difference, a result that needs confirmation in a design with comparable groups.

## 1. Introduction

Human pathophysiology is now routinely described in redox terms, and the description depends on measurement. Oxidative stress is not a single quantity. It is a set of related states distributed across compartments that exchange with each other slowly and incompletely, and any clinical study of it inherits the boundaries of the assay it uses [1,2]. Two pools can be sampled from one ordinary venous draw. The first is the extracellular thiol pool, quantified as serum thiol-disulfide homeostasis, a bulk measurement to which the single reactive cysteine-34 residue of albumin is the largest contributor [3,4]. The second is intracellular glutathione, most conveniently measured in the erythrocyte, which the serum assay does not sample at all [5].

These two pools are not the same substance observed twice. Albumin cysteine-34 is continuously replaced through hepatic synthesis, circulates in gram quantities and functions as the principal extracellular redox buffer; its adduct profile serves, in adductomic work, as an integrative record of systemic oxidant exposure [6,7]. Erythrocyte glutathione is synthesized inside a cell that has no nucleus, cannot import the tripeptide efficiently, and must maintain its own reducing capacity for about 120 days with a fixed enzymatic complement [5,8]. A sustained oxidant load might therefore leave different marks on the two pools. The prediction is straightforward. It has rarely been tested in patients.

Most clinical redox studies measure one compartment. That choice is usually made on assay availability. When both were assayed in the same subjects, they moved in the same direction with unequal amplitude. In rheumatoid arthritis, the intracellular ratio separated patients from controls more widely than the serum ratio [9]. The same asymmetry appears in Alzheimer disease [10], in small-for-gestational-age newborns [11], in hypothyroidism before and after levothyroxine replacement [12], in diabetic retinopathy [13], and in acute ischemic stroke sampled within 24 h of onset, where the glutathione system showed the largest shift [14]. If that pattern is general, it carries a methodological consequence that reaches beyond any single disease: the compartment chosen influences the effect size obtained, and a literature built on serum thiols alone would report smaller standardized differences than one built on the intracellular pool.

Acute coronary syndrome is a demanding place to test this. Ischemia and reperfusion generate reactive oxygen species in bursts, and oxidative injury is mechanistically embedded in both atherogenesis and myocardial reperfusion damage [1,15]. Non-ST-elevation myocardial infarction (NSTEMI) is also, in practice, an emergency department diagnosis. Patients arrive with undifferentiated chest pain, the diagnosis is established through serial high-sensitivity troponin sampling over the first hours [16,17,18,19], and the blood drawn at that first contact is the earliest specimen available for redox measurement. The label itself is heterogeneous: in an angiographic emergency department cohort, a proportion of patients who did not meet ST-elevation criteria nonetheless had an acutely occluded culprit artery [20]. It precedes revascularization, antiplatelet loading, statin initiation and every other intervention that perturbs redox balance. Emergency physicians hold this sampling window, and it is not recoverable later in the admission.

Serum thiol-disulfide homeostasis is well studied in this population. It is disturbed in acute myocardial infarction [21], carries prognostic information in non-ST-elevation acute coronary syndrome [22], relates to angiographic thrombus grade [23] and to inflammation in cardiac syndrome X [24], and differs by underlying coronary anatomy [25]. One group profiled S-nitrosothiol and thiol-disulfide levels together in acute coronary syndrome [26]. Routine admission indices such as the hemoglobin-to-red-cell-distribution-width ratio also carry prognostic information in acute coronary syndrome [27], but they are not measures of redox state. Every one of these studies sampled the extracellular compartment only. We have since measured both compartments together in ST-elevation myocardial infarction, where both were oxidized and the intracellular effect was the larger of the two [28]. The non-ST-elevation phenotype is different in one practical respect: the diagnosis is not immediate. It is established in the emergency department over serial troponin sampling, and the first specimen is therefore drawn while the diagnosis is still open. No report has compared the two compartments in that setting.

We measured both. Our aim was to quantify, in patients presenting to an emergency department with non-ST-elevation myocardial infarction, how the extracellular and intracellular redox compartments compare when they are assayed concurrently, and to estimate formally how large the case–control difference in each compartment is relative to the other. We did not set out to evaluate either measurement as a diagnostic test, and the design does not permit such an evaluation.

Timing matters for a redox measurement in this setting. In our cohort, the median interval between symptom onset and venepuncture was 3.5 h, which places the sample inside the window in which ischemic and early reperfusion-related oxidant production is thought to occur, and before therapy has begun to alter it. Blood drawn later in the admission carries the pharmacological history of the admission with it.

## 2. Materials and Methods

### 2.1. Study Design and Setting

This was a single-centre, cross-sectional analytical case–control study conducted in the emergency department of a university hospital in Türkiye, a tertiary referral centre. Each participant was sampled once. The manuscript follows the Strengthening the Reporting of Observational Studies in Epidemiology (STROBE) reporting guideline for observational studies [29].

### 2.2. Participants

Consecutive adults presenting to the emergency department with symptoms suggestive of acute coronary syndrome, in whom non-ST-elevation myocardial infarction was subsequently confirmed according to the Fourth Universal Definition of Myocardial Infarction [17] and who underwent coronary angiography during the index admission, were eligible. All patients presented directly to the emergency department. None was transferred from another facility.

Controls were adult volunteers attending the hospital for a general health check. They had no known chronic disease, took no regular medication and had no symptoms at the time of sampling. Their cardiovascular evaluation consisted of a clinical history and a physical examination, both of which had to be unremarkable. No electrocardiogram or echocardiogram was obtained in controls. Hypertension, diabetes, hyperlipidaemia and known coronary artery disease were therefore absent in the control group by eligibility. Current smoking was not an exclusion criterion in either group. This design produces a control group that differs from the patients in sex and in cardiovascular risk profile; the consequences are addressed in Section 2.6 and Section 4.6.

Both assays report redox state in their respective pools. Conditions that independently perturb those pools were therefore exclusion criteria applied at screening: active infection, active malignancy, chronic inflammatory or autoimmune disease, hepatic or renal dysfunction, recent transfusion, pregnancy, and current antioxidant supplementation. Participants meeting any of these were never enrolled. They are reported at the screening stage of the participant flow rather than as post-enrolment exclusions.

### 2.3. Blood Sampling and Pre-Analytical Handling

Venous blood was drawn from an antecubital vein at first medical contact in the emergency department, together with the routine admission panel, before coronary angiography and before any drug was administered in the department. Because every patient presented directly, no pre-hospital antiplatelet, anticoagulant, statin, nitrate or oxygen therapy had been given before the study sample was taken. Chronic medication taken at home before presentation (antiplatelet agents, statins and antihypertensive drugs) was recorded and is reported in Table 1. The symptom-to-sampling interval was recorded in 75 of the 82 patients, with a median of 3.5 h (interquartile range [IQR] 2.0 to 4.5). Controls were sampled under outpatient conditions, outside the emergency department. Fasting status and time of day were not recorded in either group.

Blood was collected into a plain serum tube and an ethylenediaminetetraacetic acid (EDTA) tube. Serum tubes were allowed to clot and were centrifuged at 1500× *g* for 15 min at room temperature; serum was aliquoted within 1 h of venepuncture. EDTA tubes were centrifuged immediately after venepuncture, and the deproteinized erythrocyte supernatant described in Section 2.4 was frozen within 30 min of venepuncture. Aliquots were stored at −80 °C until analysis. All samples were thawed and assayed together in a single analytical series after enrolment was complete, so each sample underwent one freeze–thaw cycle, with the same reagent lots throughout. Patient and control samples were handled by the same protocol in the same laboratory and were interleaved across analytical runs. Sample quality was assessed by visual inspection for haemolysis at the laboratory; lipaemia and icterus were not systematically assessed. Both fractions came from the same draw, so the two compartments are compared at a single time point in each participant.

### 2.4. Laboratory Analyses

All chemicals were of reagent grade and obtained from Merck (Darmstadt, Germany), and Type I ultrapure water was used throughout. Spectrophotometric analyses were performed on an ARCHITECT c8000 automated chemistry analyser (Abbott Diagnostics, Abbott Park, IL, USA).

Serum thiol-disulfide homeostasis was measured with the automated spectrophotometric method of Erel and Neselioglu [3]. Native thiol and total thiol are measured directly. Disulfide is not measured: it is derived as half the difference between total and native thiol, since reduction of one disulfide bond yields two thiol groups. The derived indices disulfide/native thiol (%) and native thiol/disulfide ratio follow from those quantities. In the assay, disulfide bonds are first reduced to free thiol groups with sodium borohydride; unused reductant is consumed with formaldehyde to prevent it from reducing the chromogenic reagent, and the total thiol content is then determined with 5,5’-dithiobis-(2-nitrobenzoic acid); native thiol is determined on an untreated aliquot in the same run. The method reports the bulk native and total thiol content of serum. Albumin cysteine-34 is the largest contributor to that pool, but the assay does not identify individual thiol species, oxidation products or covalent adducts, and its readout should not be equated with a direct measurement of albumin cysteine-34 redox status.

Erythrocyte glutathione was measured with the automated colorimetric method of Alisik, Neselioglu and Erel [30]. EDTA-anticoagulated whole blood was centrifuged at 1000× *g* for 5 min at 4 °C immediately after venepuncture. The plasma and buffy coat were discarded and replaced with an equal volume of 0.9% sodium chloride, so that packed cells and saline were present at approximately 1:1 (*v*/*v*); the cells were resuspended and centrifuged again under the same conditions (1000× *g*, 5 min, 4 °C), and this washing step was repeated three times in all, with the erythrocyte fraction retained in the tube throughout. After the final wash, the discarded saline volume was replaced with an equal volume of Type I ultrapure water, again approximately 1:1 (*v*/*v*) relative to the packed cells, to lyse the washed erythrocytes. Intracellular proteins were precipitated immediately with 20% (*w*/*v*) trichloroacetic acid at a ratio of three parts lysate to one part acid (final concentration 5%). After centrifugation, the supernatant was frozen at −80 °C within 30 min of venepuncture and stored until analysis. From venepuncture to freezing, the erythrocytes were therefore in contact with saline or water for minutes only, and the glutathione pool was fixed by acid deproteinization before storage. Reduced glutathione is measured directly. Total glutathione is measured after reduction of the oxidized form, with thiol-blocking of the reduced fraction, and oxidized glutathione is derived as half their difference; the oxidized/reduced glutathione ratio (GSSG/GSH, %) is the derived index. Measured concentrations were corrected for the dilution factors introduced during sample preparation. No normalization to hemoglobin or erythrocyte count was applied. Erythrocyte glutathione concentrations are therefore reported in µmol/L of the haemolysate prepared with this standardized volume-replacement procedure, as in our previous report with the same method [28]. The assay reports the glutathione pool of the erythrocyte lysate and nothing else. It is not a measure of intracellular redox state in other cell types or of systemic redox state.

Which quantities are measured and which are derived is stated explicitly, because the distinction determines how correlated the indices are with one another and how their uncertainty propagates. Four quantities are measured directly: native thiol, total thiol, reduced glutathione and total glutathione. The other five are arithmetic derivatives of them, and the two serum ratios are reciprocals of each other. The nine indices are therefore not independent. Their mutual correlations are given in Table S3.

### 2.5. Clinical Variables

Age, sex, hypertension, diabetes mellitus, hyperlipidaemia, known coronary artery disease and current smoking were recorded for all participants. For patients, chronic home medication, peak high-sensitivity cardiac troponin during the index admission, left ventricular ejection fraction by transthoracic echocardiography, and the Synergy between Percutaneous Coronary Intervention with TAXUS and Cardiac Surgery (SYNTAX) score derived from the index angiogram were also recorded. Troponin was measured as part of routine care in the hospital central laboratory with an Abbott high-sensitivity troponin I assay on an ARCHITECT i-series immunoassay analyser (Abbott Diagnostics, Abbott Park, IL, USA). A composite poor in-hospital outcome was defined a priori. Body mass index, renal function within the permitted range, inflammatory markers, diet, alcohol intake and menopausal status were not recorded.

### 2.6. Statistical Analysis

Analyses followed a statistical analysis plan written before the data were examined. Distributional assumptions were checked with the Shapiro–Wilk test. Normally distributed variables are summarized as mean and standard deviation and compared with the Welch *t* test, with Hedges g [31] and its 95% confidence interval (CI) as the effect size; the remainder are summarized as median and interquartile range and compared with the Mann–Whitney U test, with rank-biserial correlation as the effect size. Nine indices form the prespecified primary panel, and Benjamini–Hochberg false discovery rate q-values [32] accompany the unadjusted *p*-values for that panel. Because the nine indices are arithmetically related, the correction controls multiplicity of tests but not the dependence among them.

The contrast between compartments was treated as an estimation problem rather than an inspection of two separate effect sizes. The ratio and the difference in the absolute Hedges g values for erythrocyte reduced glutathione and serum native thiol were estimated by nonparametric bootstrap with 10,000 resamples. Cases and controls were resampled separately, and participants were resampled as units, so that the correlation between two markers measured in the same blood draw is preserved. This pairwise contrast was not part of the prespecified analysis plan. It was specified after the primary panel had been examined, and it is labelled post hoc throughout. The two markers were chosen because each is the directly measured reduced species of its compartment; standardized effects for all nine indices are presented in the Results so that the choice can be judged. The estimate pertains to this pair of measures in this sample. It does not establish the superiority of either marker in clinical or biological terms.

The imbalance between groups in age, sex, smoking and cardiovascular comorbidity was quantified with standardized mean differences for every recorded covariate [33]. Four strategies then addressed confounding. Each was applied to reduced glutathione and to native thiol with an identical covariate set. First, linear regression of each standardized marker on case status, age, sex and smoking, which yields an adjusted standardized case–control difference for each marker; the ratio and difference in the two adjusted coefficients were bootstrapped as above. Second, logistic regression of case status on each marker, per standard deviation, with the same three covariates. Third, 1:1 nearest-neighbour propensity-score matching on age, sex and smoking, with a calliper of 0.2 standard deviations of the logit propensity score. For the matched analysis, we report the propensity-score distributions, the number of participants left unmatched, and covariate balance before and after matching [33]. Fourth, restriction to participants free of every recorded cardiovascular risk factor, including smoking. Hypertension, diabetes, hyperlipidaemia and known coronary disease were absent in all controls by eligibility. There is no common support for these variables. They cannot be adjusted for by any method, and the four strategies address only the three covariates that were measured in both groups.

At the request of peer review, we also report, as an exploratory analysis, the area under the receiver operating characteristic curve for each index with its 95% confidence interval, and pairwise comparisons of correlated areas by the DeLong method [34]. These analyses were fitted and evaluated in the same sample, without prespecified thresholds or external validation. They describe case–control separation in this dataset only. They support no diagnostic claim.

Associations between redox indices and clinical severity within the patient group used the Spearman coefficient, with 95% confidence intervals from the Fisher z transformation. All severity analyses are exploratory. Benjamini–Hochberg q values were calculated within each severity measure, that is, across the nine indices tested against that measure (nine tests per family). Whether an index was associated more strongly with one severity measure than with another was not formally tested. Analyses were performed in IBM SPSS Statistics for Macintosh, version 29.0 (IBM Corp., Armonk, NY, USA) and in R version 4.6.1 (R Foundation for Statistical Computing, Vienna, Austria) within RStudio version 2026.09.0+174 (Posit Software, PBC, Boston, MA, USA), using the packages effectsize 1.0.3, pROC 1.19.0.1 and boot 1.3-32; the bootstrap comparisons, the adjusted models, the propensity-score matching and the receiver operating characteristic analyses were run in R. The analysis code is available from the corresponding author on request.

### 2.7. Ethics

The study was approved by the institutional Clinical Research Ethics Committee (decision 2023/220, dated 20 June 2023) and conducted in accordance with the Declaration of Helsinki. Written informed consent for participation was obtained from every participant before enrolment; the consent form was distributed to, and signed by, every participant.

## 3. Results

### 3.1. Participant Flow and Baseline Characteristics

**Figure 1:**
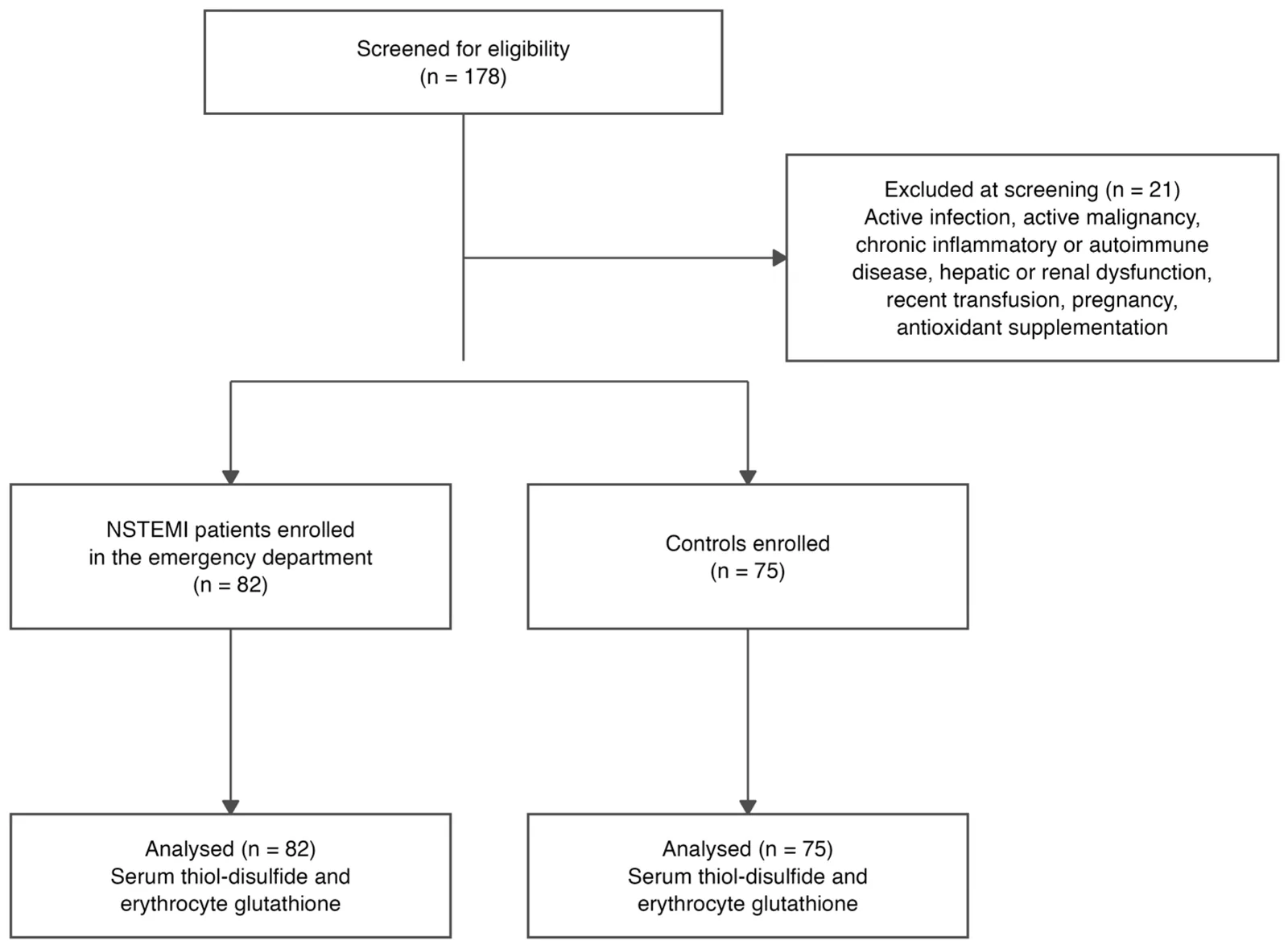
Participant flow. Patients meeting an eligibility exclusion criterion at screening were never enrolled and are shown at the screening stage. NSTEMI, non-ST-elevation myocardial infarction.

Of 178 individuals screened, 157 were analyzed: 82 patients with non-ST-elevation myocardial infarction and 75 controls (Figure 1). Patients were older, with a median age of 59.0 years (IQR 48.2 to 67.0) against 54.0 years (45.0 to 62.0) in controls (*p* = 0.012), and predominantly male, 65 of 82 (79.3%) against 21 of 75 (28.0%) (*p* < 0.001). Hypertension, diabetes, hyperlipidaemia and known coronary artery disease were present only among patients, as the eligibility criteria require. Before presentation, 32 patients (39.0%) were taking an antiplatelet agent, 38 (46.3%) a statin and 53 (64.6%) an antihypertensive drug. Baseline characteristics are given in Table 1. The standardized mean differences between groups were 0.47 for age, 1.20 for male sex and 0.74 for current smoking; for the four comorbidities, the difference is undefined in the usual sense because the control prevalence is zero (Table S1).

### 3.2. Case–Control Differences in the Two Compartments

**Figure 2:**
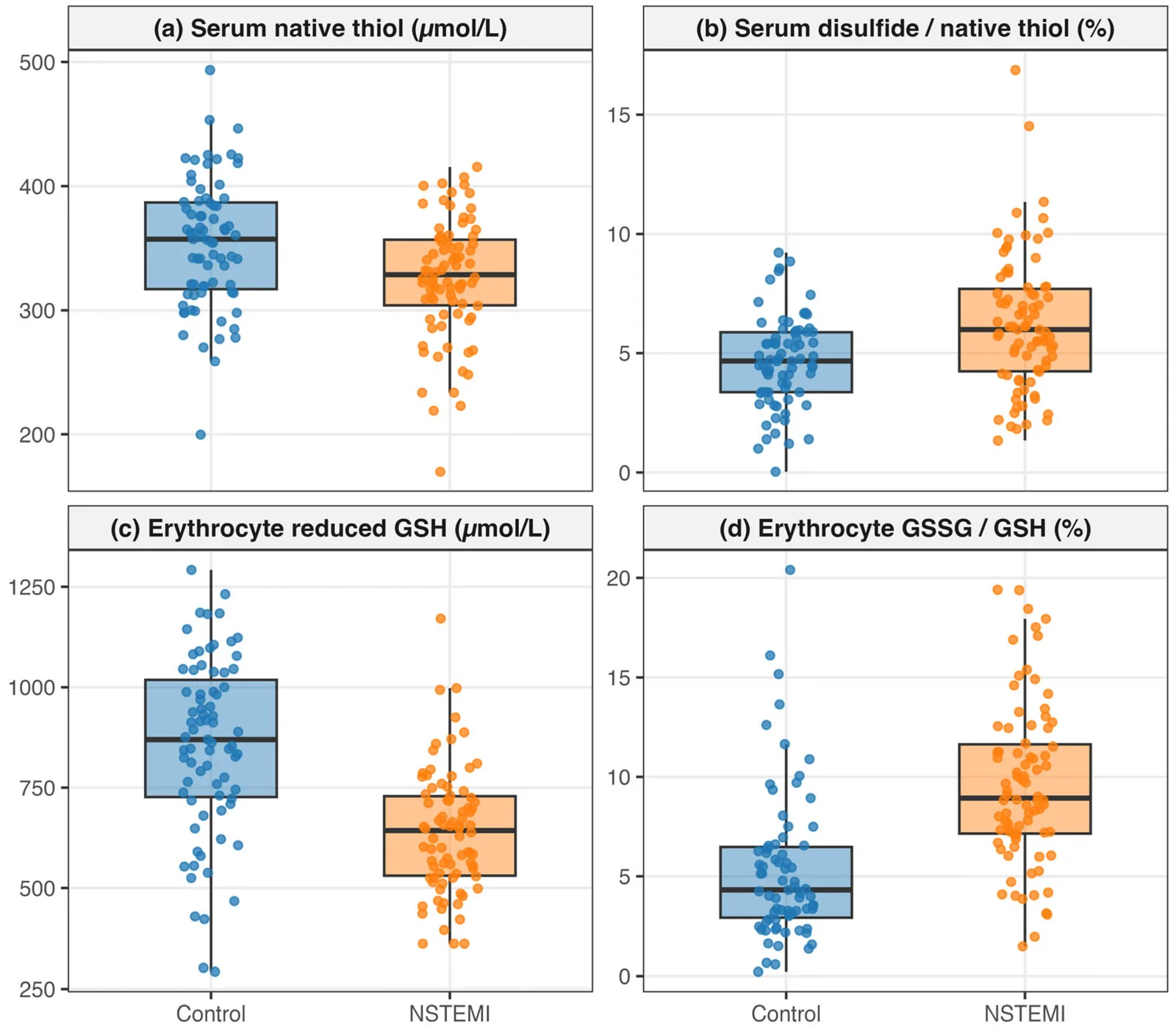
Distribution of the four principal redox indices by group. (**a**) Serum native thiol (measured); (**b**) serum disulfide/native thiol ratio (derived); (**c**) erythrocyte reduced glutathione (measured); (**d**) erythrocyte GSSG/GSH ratio (derived). Boxes show the median and interquartile range, whiskers extend to 1.5 times the interquartile range, and individual participants are plotted. Controls are shown in blue and patients with NSTEMI in orange. GSH, reduced glutathione; GSSG, oxidized glutathione; NSTEMI, non-ST-elevation myocardial infarction.

Every index in the primary panel differed between groups, and every difference ran in the direction consistent with greater oxidation in the NSTEMI group (Table 2, Figure 2). In the extracellular compartment, native thiol was 326.0 ± 47.6 µmol/L in patients against 353.8 ± 51.5 µmol/L in controls (g = −0.56, 95% CI −0.88 to −0.24; *p* < 0.001), total thiol was 364.9 ± 46.0 against 386.0 ± 53.3 µmol/L (g = −0.42, −0.74 to −0.11), and the derived disulfide was 19.5 ± 7.0 against 16.1 ± 6.1 µmol/L (g = 0.51, 0.19 to 0.82). The derived disulfide/native thiol ratio was 5.99% (IQR 4.24 to 7.69) against 4.67% (3.37 to 5.88), rank-biserial r = 0.34 (0.17 to 0.51).

The intracellular compartment showed the larger standardized differences. Reduced glutathione was 640.0 ± 153.1 µmol/L in patients against 855.0 ± 219.2 µmol/L in controls (g = −1.14, −1.48 to −0.80; *p* < 0.001, q < 0.001), total glutathione 758.7 ± 170.6 against 935.0 ± 212.0 µmol/L (g = −0.92, −1.25 to −0.59), and the derived oxidized glutathione 59.4 ± 23.7 against 40.0 ± 18.8 µmol/L (g = 0.90, 0.57 to 1.23). The derived oxidized/reduced ratio was 8.93% (7.15 to 11.64) against 4.32% (2.93 to 6.48), r = 0.61 (0.46 to 0.74), *p* < 0.001. The nine indices are strongly interrelated. Within each compartment, the measured pair correlated at Spearman rho 0.96 (native and total thiol) and 0.98 (reduced and total glutathione), and the derived indices correlated at 0.87 to 1.00 with the measured quantities they are calculated from (Table S3).

### 3.3. Comparison of the Two Compartments

**Figure 3:**
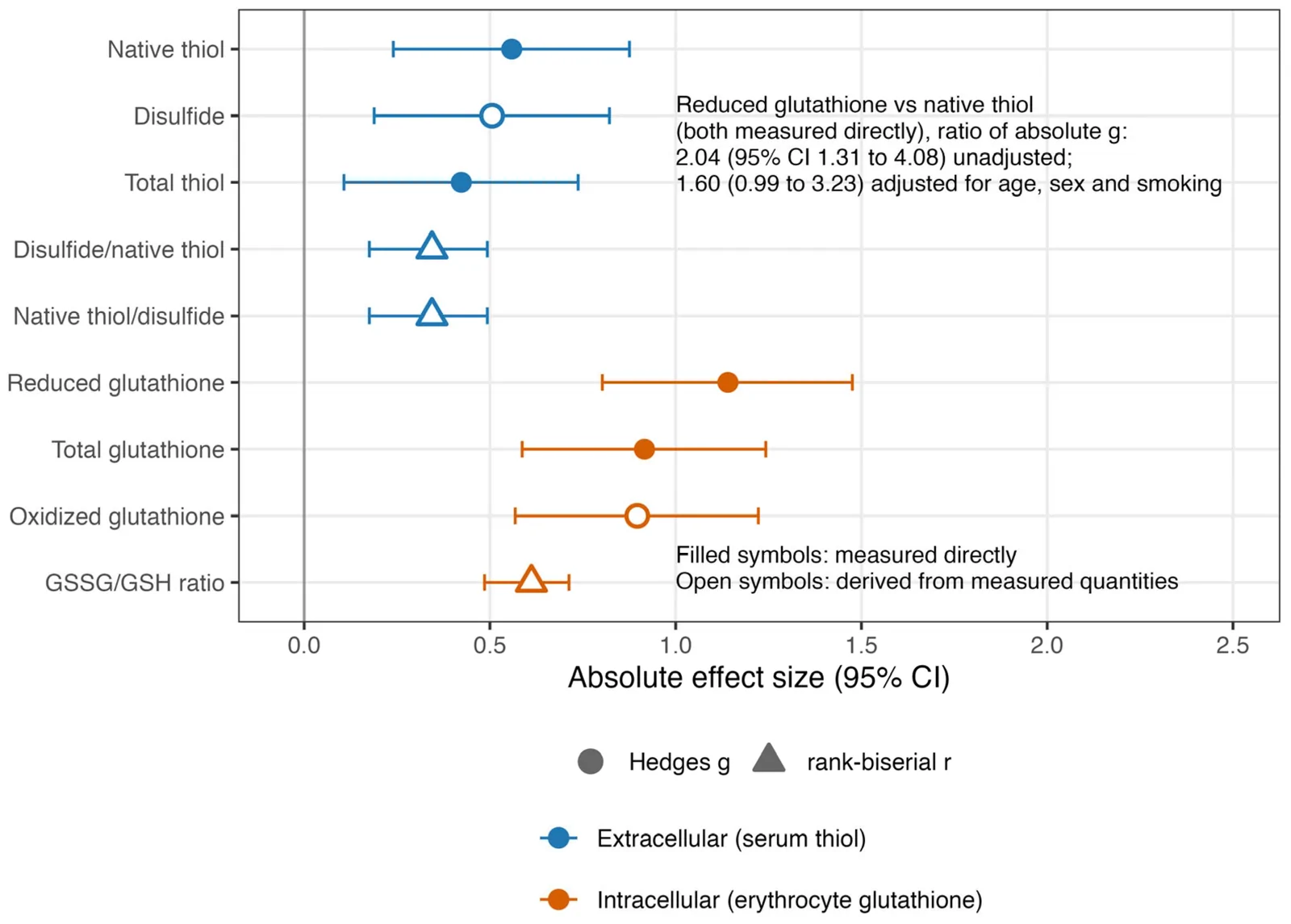
Absolute unadjusted effect sizes for the nine redox indices, with 95% confidence intervals, grouped by compartment. Circles are Hedges g and triangles are rank-biserial correlations, which are not on the same scale and are therefore not directly comparable with one another. Filled symbols mark quantities measured directly by the assay and open symbols mark derived quantities. The annotation gives the post hoc bootstrap comparison of the two directly measured reduced-form markers, before and after adjustment for age, sex and smoking. CI, confidence interval; GSSG/GSH, ratio of oxidized to reduced glutathione.

Comparing the two unadjusted effect sizes directly, the absolute Hedges g was 1.14 for erythrocyte reduced glutathione and 0.56 for serum native thiol. The bootstrap ratio of the two was 2.04 (95% CI 1.31 to 4.08) and the difference was 0.58 (0.22 to 0.99; bootstrap *p* = 0.002). The interval excludes unity, so the wider separation in the intracellular compartment is unlikely to be sampling variation alone, although its upper bound leaves the magnitude imprecisely estimated (Figure 3). This contrast was specified after the primary panel had been examined. It is post hoc. It describes the size of the case–control difference in this sample, not the diagnostic or biological value of either measurement.

### 3.4. Group Imbalance and Adjusted Comparisons

Table 3 sets out the two measured markers side by side under each confounding strategy, with the same covariates for both. In linear models adjusted for age, sex and smoking, the standardized case–control difference was −0.97 (95% CI −1.31 to −0.63) for reduced glutathione and −0.60 (−0.98 to −0.22) for native thiol. The ratio of the adjusted coefficients was 1.60 (0.99 to 3.23) and their difference 0.36 (−0.01 to 0.74; bootstrap *p* = 0.054). Adjustment therefore reduced the asymmetry from an estimated twofold to an estimated 1.6-fold. The adjusted interval reaches unity. Adjusted estimates for all nine indices are given in Table S2, and Figure S3 displays the unadjusted and adjusted standardized differences for all nine indices on a common scale. In logistic models with the same covariates, the odds of NSTEMI per standard deviation were 0.30 (0.18 to 0.50) for reduced glutathione and 0.50 (0.31 to 0.79) for native thiol; the GSSG/GSH ratio gave 1.33 per percentage point (1.17 to 1.50; *p* < 0.001), as in the original submission.

Propensity scores estimated from age, sex and smoking overlapped only partly. The median score was 0.76 (IQR 0.54 to 0.90) in patients and 0.23 (0.16 to 0.55) in controls. The region of common support ran from 0.15 to 0.91, leaving 13 patients above it and 18 controls below it (Figure S1). Matching within a calliper of 0.31 logit units produced 38 pairs and discarded 44 patients and 37 controls. Matching balanced the three modelled covariates, with standardized mean differences after matching of −0.18 for age, 0.11 for male sex and −0.06 for smoking. It could not balance the four comorbidities, which remained absent in matched controls (Table S1). In the matched pairs, reduced glutathione gave g = −1.14 (−1.76 to −0.66) and native thiol g = −0.56 (−1.04 to −0.13). The ratio was 2.03 (0.97 to 7.39).

Restriction to the 67 participants with no recorded cardiovascular risk factor, including smoking, left 7 patients and 60 controls. In this subgroup, reduced glutathione gave g = −1.11 (−1.75 to −0.63) and native thiol g = −1.15 (−1.85 to −0.62), a ratio of 0.96 (0.62 to 1.56). Both compartments separated the groups. The asymmetry between them was not present. With 7 patients, this subgroup cannot be read as a confirmatory analysis in either direction. Within the patient group, no recorded risk factor was associated with the GSSG/GSH ratio or with reduced glutathione at a standardized mean difference above 0.31. That argues against a single comorbidity driving the intracellular difference, but given the sample size and the correlation among comorbidities, it does not exclude confounding.

### 3.5. Exploratory Discrimination Analysis

Areas under the receiver operating characteristic curve followed the ranking of the standardized differences (Table S4, Figure S2). They were 0.80 (95% CI 0.72 to 0.87) for reduced glutathione and 0.81 (0.73 to 0.88) for the GSSG/GSH ratio, against 0.65 (0.56 to 0.73) for native thiol and 0.67 (0.59 to 0.76) for the disulfide/native thiol ratio. The paired difference in area between reduced glutathione and native thiol was 0.15 (DeLong *p* = 0.001), and between the two ratios 0.13 (*p* = 0.006). These values describe separation of the groups as they were selected. For comparison, the presence of any of the four comorbidities, which were absent in controls by eligibility, separated the groups with an area of 0.93 (0.89 to 0.97) without any laboratory measurement. Discrimination in this design therefore reflects participant selection as much as redox biology. None of these areas should be read as diagnostic accuracy.

### 3.6. Exploratory Associations with Clinical Severity

Within the patient group, the associations with severity differed between compartments (Table 4). The SYNTAX score was associated with the disulfide/native thiol ratio (rho = 0.47, 95% CI 0.28 to 0.62; q < 0.001), with disulfide (rho = 0.40, 0.20 to 0.57; q < 0.001) and inversely with native thiol (rho = −0.25, −0.45 to −0.04; q = 0.048). It was not associated with any glutathione index (reduced glutathione rho = −0.21, −0.41 to 0.01; q = 0.11). Left ventricular ejection fraction, median 50% (IQR 45 to 55), was associated with reduced glutathione (rho = 0.30, 0.09 to 0.49; q = 0.05) and total glutathione (rho = 0.28, 0.07 to 0.47; q = 0.05) and with no serum index. Peak troponin was associated with no redox index (minimum unadjusted *p* = 0.29) and not with ejection fraction (rho = −0.08, −0.29 to 0.14). The coefficients are modest. The analyses are exploratory, and the difference between compartments in these associations was not formally tested. Eight patients met the composite poor in-hospital outcome; their descriptive comparison with the remaining 74 is hypothesis-generating only.

## 4. Discussion

### 4.1. Principal Findings

Measured in the same blood sample, in patients whose myocardial infarction had just been diagnosed in an emergency department, both redox compartments differed from controls in the direction of greater oxidation. The standardized case–control difference was larger for erythrocyte reduced glutathione than for serum native thiol. The unadjusted ratio of the two effect sizes was 2.04 (95% CI 1.31 to 4.08). After adjustment for age, sex and smoking, the ratio was 1.60, with an interval that reaches unity (0.99 to 3.23). In propensity-matched pairs, it was 2.03 with a wide interval. In the small subgroup free of recorded risk factors, the two markers separated the groups equally. The point estimates consistently favour the intracellular compartment, but their precision is low, and the design cannot separate the contribution of the disease from that of the risk profile that accompanied it. The result is therefore a description of what two assays showed in one highly imbalanced case–control sample. It is not a demonstration that one assay is the more sensitive measure of oxidative stress.

### 4.2. Possible Reasons for the Unequal Separation

Compartment physiology offers a hypothesis. The two pools are renewed on different timescales. Serum thiol is dominated by albumin, replenished continuously by hepatic synthesis and buffered by a circulating pool measured in grams [3,6]. Oxidation of cysteine-34 is reversible, and the pool is refreshed on the timescale of albumin turnover; adduct-level analysis of the residue exists precisely because the bulk thiol measurement averages over this exchange [7]. Erythrocyte glutathione behaves differently. The mature red cell is anucleate, cannot import glutathione at any useful rate and depends on a fixed complement of synthetic and recycling enzymes across a lifespan of roughly 120 days [5,8]. On this reading, a sustained oxidant load would deplete the intracellular pool and the pool would recover slowly, while the extracellular compartment would be held closer to a set point.

The present data are consistent with that hypothesis but do not test it. Blood was collected at a median of 3.5 h after symptom onset. There was no pre-event baseline, no serial sampling and no follow-up, so the temporal window that each measurement integrates over cannot be determined here. The absence of a correlation between either compartment and peak troponin does not establish that the glutathione difference is chronic or unrelated to the acute episode. Troponin is an imperfect proxy for oxidant exposure; it is affected by timing, infarct size, reperfusion, renal function and management. Likewise, the absence of an association between individual risk factors and the intracellular indices within 82 patients does not exclude confounding, given the limited power and the correlation among comorbidities. Whether depleted erythrocyte glutathione in these patients preceded the coronary event, arose from it, or reflects the risk profile that accompanied it is a question for a study with pre-event or serial samples. Oxidative stress markers in acute infarction are known to differ by underlying coronary anatomy despite comparable inflammatory status [25]. That observation is compatible with more than one of these explanations.

### 4.3. The Two Compartments May Carry Different Clinical Information

The exploratory severity analyses point to a possibility that goes beyond a ranking of effect sizes. Within patients, the serum indices were associated with angiographic burden, with the largest coefficient for the disulfide/native thiol ratio against the SYNTAX score, while the glutathione indices were not. Ejection fraction showed a different pattern. It was associated with reduced and total glutathione and with no serum index. The coefficients are modest, the comparison between compartments was not formally tested, and the pattern needs replication. If it holds, the compartments report different aspects of the same illness rather than one quantity twice, and a study that samples only one of them loses the other, whichever it picks. This possibility is easy to miss in a design that measures a single pool, which is how nearly all of the existing acute coronary literature is built [21,22,23,24,26].

#### The Erythrocyte as a Candidate Sentinel in Acute Coronary Syndrome

There is a specific reason to expect the red cell to be informative here rather than incidentally different. Erythrocytes traverse the coronary microcirculation repeatedly and are exposed directly to the oxidant burst that accompanies ischemia and reperfusion, while carrying no capacity for transcriptional response [5,15]. The mature cell cannot replace damaged proteins over its lifespan of about 120 days. Its redox phenotype is therefore read by some authors as an integrated record of cumulative systemic exposure rather than a snapshot of the extracellular milieu at the moment of sampling [5,35]. Oxidation sustained there persists until the cell is cleared. The extracellular pool, by contrast, is under continuous hepatic resupply, so an equivalent oxidant exposure might produce a smaller measured deflection. The hypothesis that follows is that the two compartments differ in what they integrate over rather than in what they detect: hours for serum thiol, weeks for erythrocyte glutathione. The present design cannot test that hypothesis, and we present it as a direction for serial-sampling studies rather than as a conclusion. The exploratory observation that glutathione was associated with ejection fraction while serum indices were associated with angiographic burden would be compatible with such a division, since ventricular performance reflects accumulated myocardial injury and the SYNTAX score describes the current anatomical state of the vessels.

Chronic exposures that deplete glutathione slowly would be expected to act on the intracellular pool preferentially. Cigarette smoking imposes exactly this kind of sustained oxidant load [36], and hyperglycaemia has comparable effects on redox balance in diabetes [37]. Both were more common among our patients, and the analyses in Section 3.4 do not exclude their contribution. Adjustment for age, sex and smoking reduced the ratio of effect sizes from 2.04 to 1.60 with an interval that reaches unity, and in the seven patients free of recorded risk factors the two compartments separated the groups equally. Smoking and the comorbidities concentrated in the patient group therefore remain candidate contributors to the asymmetry, and the present data cannot apportion it between the disease and the risk profile.

### 4.4. Consistency Across Disease Settings

The direction we observed matches every published dataset in which both compartments were measured with these two automated assays in the same participants. Rheumatoid arthritis showed the wider separation intracellularly [9]. So did Alzheimer disease [10], small-for-gestational-age newborns [11], hypothyroidism [12] and diabetic retinopathy [13]. Those settings differ in mechanism, in time course, and in the population studied, and the pattern still appears. Earlier work with other analytical methods points the same way. Paşaoğlu and colleagues measured erythrocyte glutathione alongside plasma total thiols and lipid peroxidation markers in type 2 diabetes [38], and Murakami and colleagues described impaired erythrocyte glutathione metabolism, with a shift toward the oxidized form, in diabetes decades before automated paired assays were available [39]. Those studies did not use the paired methodology applied here, so their effect sizes are not directly comparable with ours, but they show that the erythrocyte and the plasma or serum pool were treated as distinct redox compartments long before paired assays existed. Acute coronary syndrome now extends the paired-assay evidence to an event measured within hours of onset. In a separate cohort of 90 patients with ST-elevation myocardial infarction and 79 controls, sampled at first contact with the same two assays, both compartments were again oxidized and the intracellular effect was again the larger of the two, with Hedges g of −1.58 for reduced glutathione against −1.38 for native thiol, although the margin between them was narrower than the one reported here [28]. In acute ischemic stroke, measured concurrently with the same two assays in 85 patients sampled within 24 h of onset and 79 controls, both systems again shifted toward oxidation and the glutathione system shifted most, with a rank-biserial correlation of 0.88 for the GSSG/GSH ratio [14]. Single-compartment measurement remains the norm across acute illness: thiol levels in COVID-19 [40], thiol-disulfide homeostasis in sepsis [41], and enzymatic and non-enzymatic redox markers after acute ischemic stroke [42]. Within this journal, other groups characterized thiol-disulfide homeostasis in lung cancer [43] and, in this Special Issue, systemic oxidative and nitrosative stress in benign prostatic hyperplasia [44]. All of these sample a single compartment. The present data indicate that the choice between compartments is not neutral, and they show what is given up by making it without comparison.

### 4.5. Implications for Study Design and for Acute Care

For investigators, the practical implication concerns design. In this sample, and in the earlier two-compartment datasets, the intracellular measurement gave the larger standardized case–control difference; if that holds in designs with comparable groups, it affects the number of participants a study of cumulative redox burden requires. If the question concerns vascular disease burden, the serum indices carried that association here and glutathione did not, although that observation is exploratory. Studies that measure one compartment and generalize to redox state as a whole are making an assumption this dataset does not support.

For acute care, the relevant point is access. The sample analyzed here was drawn at first medical contact, before angiography and before any drug that alters redox balance. That specimen exists once. It cannot be recovered later in the admission. Emergency physicians hold that window, and redox studies of acute illness depend on it, whether or not the resulting paper is framed as emergency medicine. Both assays run on an ordinary venous sample, are automated and are inexpensive, so adding the intracellular measurement to an emergency department study costs one additional aliquot and no additional venipuncture.

Therapeutic implications are indirect and should be read cautiously. N-acetylcysteine regenerates albumin cysteine-34 in vivo [45], and trials have evaluated it as an adjunct during coronary reperfusion [46]. Whether the intracellular pool responds comparably is unresolved, and our cross-sectional design cannot address it.

### 4.6. Limitations

The principal limitation is that the comparison groups were not comparable. Patients were predominantly male and controls predominantly female. Patients were older and smoked more often, and the four classical cardiovascular risk factors were absent in controls by eligibility (standardized mean differences 0.47 to 1.20 for the measured covariates, Table S1). This creates structural non-overlap. Hypertension, diabetes, hyperlipidaemia and known coronary disease cannot be adjusted for by any method. Adjustment for age, sex and smoking does not resolve confounding by these conditions or by medication history, body mass index, diet, alcohol intake, renal function within the permitted range, inflammatory status or menopausal status, none of which was recorded. Residual confounding therefore remains possible, and the adjusted ratio should not be read as an estimate free of it. Propensity-score matching on three covariates balanced those three covariates and nothing else; it discarded more than half of each group, and it does not restore exchangeability. The risk-factor-free subgroup contained 7 patients. It cannot be read as confirmatory in either direction. The adjusted ratio of effect sizes reached unity, so the asymmetry between compartments should be regarded as an observation in need of confirmation in an age-matched and sex-matched design with comparable risk profiles, as recent work in this journal on a comparable case–control design has also concluded [44].

The pairwise contrast between reduced glutathione and native thiol was not prespecified and is post hoc. The nine indices are arithmetically interrelated (Table S3). The panel therefore does not provide nine independent confirmations, and the false discovery rate correction addresses the number of tests rather than their dependence. Both measurements are assay-specific. The thiol method reports the bulk native and total thiol content of serum, not albumin cysteine-34 specifically, and the glutathione method reports the erythrocyte lysate pool. The two are not general measures of oxidative stress. The discrimination analyses were exploratory and describe separation of selected groups rather than diagnostic performance. The design is cross-sectional and single-centre, with no pre-event baseline and no serial sampling, so the temporal window that each compartment integrates over, and the trajectory of either after the index event, are unknown. Fasting status and time of day were not recorded. Controls were sampled under outpatient conditions while patients were sampled in the emergency department, and lipaemia and icterus were not systematically assessed. The composite poor-outcome subgroup contained 8 patients, and all correlation and subgroup analyses are exploratory.

## 5. Conclusions

In this single-centre case–control study of patients diagnosed with non-ST-elevation myocardial infarction at emergency department presentation, both the serum thiol-disulfide and the erythrocyte glutathione indices differed from controls in the direction of greater oxidation. Of the two directly measured reduced-form markers, erythrocyte reduced glutathione showed the larger standardized case–control difference; this comparison was post hoc. The bootstrap ratio was 2.04 (95% CI 1.31 to 4.08) before and 1.60 (0.99 to 3.23) after adjustment for age, sex and smoking. The groups differed substantially in sex and cardiovascular risk profile, and the asymmetry was not seen in the small risk-factor-free subgroup. The finding therefore describes this sample and requires confirmation in comparable groups. In exploratory analyses, the two compartments were associated with different severity measures. Each assay reports its own pool, not a global measure of oxidative stress. Within those limits, the compartment a clinical redox study chooses to sample influenced the size of the difference it observed.

## Figures and Tables

**Table 1:** Baseline characteristics of patients with non-ST-elevation myocardial infarction and controls.

Characteristic	NSTEMI (*n* = 82)	Controls (*n* = 75)
Age, years	59.0 (48.2 to 67.0)	54.0 (45.0 to 62.0)
Male sex	65 (79.3)	21 (28.0)
Current smoking	44 (53.7)	15 (20.0)
Hypertension	50 (61.0)	0 (0)
Diabetes mellitus	19 (23.2)	0 (0)
Hyperlipidaemia	34 (41.5)	0 (0)
Known coronary artery disease	23 (28.0)	0 (0)
Antiplatelet agent †	32 (39.0)	0 (0)
Statin †	38 (46.3)	0 (0)
Antihypertensive drug †	53 (64.6)	0 (0)
Symptom-to-sampling interval, h *	3.5 (2.0 to 4.5)	not applicable
Peak cardiac troponin, ng/L	763 (242 to 2390)	not applicable
Left ventricular ejection fraction, %	50 (45 to 55)	not applicable
SYNTAX score	18.0 (12.0 to 23.0)	not applicable

Continuous variables are median (interquartile range); categorical variables are *n* (%). * Recorded in 75 of 82 patients. † Chronic medication taken at home before presentation; controls were eligible only if they took no regular medication. NSTEMI, non-ST-elevation myocardial infarction; SYNTAX, Synergy between Percutaneous Coronary Intervention with TAXUS and Cardiac Surgery.

**Table 2:** Serum thiol-disulfide and erythrocyte glutathione indices by group.

Index	Type	NSTEMI (*n* = 82)	Controls (*n* = 75)	Effect Size (95% CI)	*p*	q *
Native thiol, µmol/L	Measured	326.0 ± 47.6	353.8 ± 51.5	g = −0.56 (−0.88 to −0.24)	<0.001	<0.001
Total thiol, µmol/L	Measured	364.9 ± 46.0	386.0 ± 53.3	g = −0.42 (−0.74 to −0.11)	0.009	0.009
Disulfide, µmol/L	Derived	19.5 ± 7.0	16.1 ± 6.1	g = 0.51 (0.19 to 0.82)	0.002	0.002
Disulfide/native thiol, %	Derived	5.99 (4.24 to 7.69)	4.67 (3.37 to 5.88)	r = 0.34 (0.18 to 0.49)	<0.001	<0.001
Native thiol/disulfide ratio	Derived	16.69 (13.00 to 23.57)	21.42 (17.01 to 29.69)	r = −0.34 (−0.49 to −0.18)	<0.001	<0.001
Reduced glutathione, µmol/L	Measured	640.0 ± 153.1	855.0 ± 219.2	g = −1.14 (−1.48 to −0.80)	<0.001	<0.001
Total glutathione, µmol/L	Measured	758.7 ± 170.6	935.0 ± 212.0	g = −0.92 (−1.24 to −0.59)	<0.001	<0.001
Oxidized glutathione, µmol/L	Derived	59.4 ± 23.7	40.0 ± 18.8	g = 0.90 (0.57 to 1.22)	<0.001	<0.001
GSSG/GSH, %	Derived	8.93 (7.15 to 11.64)	4.32 (2.93 to 6.48)	r = 0.61 (0.49 to 0.71)	<0.001	<0.001

Values are mean ± standard deviation for variables compared with the Welch *t* test and median (interquartile range) for variables compared with the Mann–Whitney U test. Effect sizes are Hedges g for the former and rank-biserial correlation r for the latter. Measured, quantity measured directly by the assay; Derived, quantity calculated from measured quantities. Erythrocyte glutathione is expressed per litre of haemolysate (Section 2.4). CI, confidence interval; GSSG/GSH, ratio of oxidized to reduced glutathione; NSTEMI, non-ST-elevation myocardial infarction. * Benjamini–Hochberg false discovery rate across the nine-index panel.

**Table 3:** Case–control difference in reduced glutathione and native thiol under each confounding strategy, with identical covariates for both markers.

Strategy	*n*	Reduced Glutathione, |Effect| (95% CI)	Native Thiol, |Effect| (95% CI)	Ratio (95% CI)	Difference (95% CI)
Unadjusted, Hedges g	157	1.14 (0.80 to 1.48)	0.56 (0.24 to 0.88)	2.04 (1.31 to 4.08)	0.58 (0.22 to 0.99)
Adjusted for age, sex and smoking, β	157	0.97 (0.63 to 1.31)	0.60 (0.23 to 0.98)	1.60 (0.99 to 3.23)	0.36 (−0.01 to 0.74)
Propensity-score matched on age, sex and smoking, Hedges g	38 pairs	1.14 (0.66 to 1.76)	0.56 (0.13 to 1.04)	2.03 (0.97 to 7.39)	0.58 (−0.02 to 1.29)
Restricted to participants free of all recorded risk factors, Hedges g	67 (7 patients, 60 controls)	1.11 (0.63 to 1.75)	1.15 (0.62 to 1.85)	0.96 (0.62 to 1.56)	−0.04 (−0.57 to 0.50)

g, Hedges g; CI, confidence interval; β, standardized coefficient for case status from a linear model of the standardized marker on group, age, sex and current smoking. Absolute values are shown. The ratio and difference are |effect for reduced glutathione| relative to |effect for native thiol|; their intervals are percentile bootstrap intervals from 10,000 resamples (participants resampled as units; matched pairs resampled as units in the matched analysis). The pairwise contrast was not prespecified.

**Table 4:** Spearman correlations between redox indices and measures of disease severity in the 82 patients (exploratory).

Severity Measure	Index	Type	rho (95% CI)	*p*	q
SYNTAX score	Native thiol	Measured	−0.25 (−0.45 to −0.04)	0.021	0.048
SYNTAX score	Total thiol	Measured	−0.12 (−0.33 to 0.10)	0.279	0.358
SYNTAX score	Disulfide	Derived	0.40 (0.20 to 0.57)	<0.001	<0.001
SYNTAX score	Disulfide/native thiol	Derived	0.47 (0.28 to 0.62)	<0.001	<0.001
SYNTAX score	Native thiol/disulfide ratio	Derived	−0.47 (−0.62 to −0.28)	<0.001	<0.001
SYNTAX score	Reduced glutathione	Measured	−0.21 (−0.41 to 0.01)	0.059	0.106
SYNTAX score	Total glutathione	Measured	−0.17 (−0.37 to 0.05)	0.136	0.203
SYNTAX score	Oxidized glutathione	Derived	−0.03 (−0.25 to 0.19)	0.792	0.792
SYNTAX score	GSSG/GSH	Derived	0.08 (−0.14 to 0.29)	0.478	0.538
Peak cardiac troponin	Native thiol	Measured	0.00 (−0.22 to 0.22)	0.996	0.996
Peak cardiac troponin	Total thiol	Measured	−0.01 (−0.23 to 0.20)	0.911	0.996
Peak cardiac troponin	Disulfide	Derived	−0.08 (−0.29 to 0.14)	0.484	0.918
Peak cardiac troponin	Disulfide/native thiol	Derived	−0.07 (−0.28 to 0.15)	0.547	0.918
Peak cardiac troponin	Native thiol/disulfide ratio	Derived	0.07 (−0.15 to 0.28)	0.547	0.918
Peak cardiac troponin	Reduced glutathione	Measured	0.02 (−0.20 to 0.24)	0.851	0.996
Peak cardiac troponin	Total glutathione	Measured	0.06 (−0.16 to 0.27)	0.612	0.918
Peak cardiac troponin	Oxidized glutathione	Derived	0.11 (−0.11 to 0.32)	0.315	0.918
Peak cardiac troponin	GSSG/GSH	Derived	0.12 (−0.10 to 0.33)	0.293	0.918
Left ventricular ejection fraction	Native thiol	Measured	0.11 (−0.11 to 0.32)	0.325	0.653
Left ventricular ejection fraction	Total thiol	Measured	0.12 (−0.10 to 0.33)	0.286	0.653
Left ventricular ejection fraction	Disulfide	Derived	0.00 (−0.22 to 0.22)	0.996	0.996
Left ventricular ejection fraction	Disulfide/native thiol	Derived	−0.07 (−0.29 to 0.15)	0.508	0.653
Left ventricular ejection fraction	Native thiol/disulfide ratio	Derived	0.07 (−0.15 to 0.29)	0.508	0.653
Left ventricular ejection fraction	Reduced glutathione	Measured	0.30 (0.09 to 0.49)	0.006	0.050
Left ventricular ejection fraction	Total glutathione	Measured	0.28 (0.07 to 0.47)	0.011	0.050
Left ventricular ejection fraction	Oxidized glutathione	Derived	−0.01 (−0.22 to 0.21)	0.941	0.996
Left ventricular ejection fraction	GSSG/GSH	Derived	−0.09 (−0.30 to 0.13)	0.416	0.653

rho, Spearman correlation coefficient with 95% confidence interval from the Fisher z transformation; *p*, unadjusted; q, Benjamini–Hochberg false discovery rate calculated within each severity measure (nine tests per family). Measured, quantity measured directly; Derived, quantity calculated from measured quantities. CI, confidence interval; GSSG/GSH, ratio of oxidized to reduced glutathione; SYNTAX, Synergy between Percutaneous Coronary Intervention with TAXUS and Cardiac Surgery.

## Data Availability

The data presented in this study are available on request from the corresponding author. The data are not publicly available because the ethics approval obtained for this study does not cover public deposition of individual patient data.

## Supplementary Materials

The following supporting information can be downloaded at: https://www.mdpi.com/article/10.3390/antiox15101334/s1, Table S1: Covariate balance before and after 1:1 propensity-score matching on age, sex and current smoking (calliper 0.2 standard deviations of the logit propensity score); Table S2: Unadjusted and adjusted standardized case–control differences for all nine redox indices (linear models of each standardized index on case status, with and without age, sex and current smoking; *n* = 157); Table S2b: Adjusted odds of non-ST-elevation myocardial infarction per one standard deviation of the index (logistic regression with age, sex and current smoking; *n* = 157; Wald 95% confidence intervals); Table S3: Spearman correlation matrix among the nine redox indices in all 157 participants; Table S4: Exploratory discrimination between patients and controls: area under the receiver operating characteristic curve (AUC) for each index, with DeLong 95% confidence intervals (*n* = 157); Table S4b: Pairwise comparison of correlated AUCs by the DeLong method; Figure S1: Propensity-score distributions (age, sex and current smoking) in patients with non-ST-elevation myocardial infarction (above the axis) and controls (below the axis); Figure S2: Receiver operating characteristic curves for the four directly measured indices and the two principal ratios (exploratory; fitted and evaluated in the same sample); Figure S3: Unadjusted and adjusted standardized case–control differences for all nine redox indices on a common scale (β from linear models of each standardized index on case status, without and with age, sex and current smoking; *n* = 157; bars are 95% confidence intervals; negative values indicate lower values in patients).
