# Supplementary material for: Erythrocyte Glutathione Shows a Larger Case–Control Difference than Serum Thiol-Disulfide Homeostasis at Emergency Department Presentation with Non-ST-Elevation Myocardial Infarction
Source: Antioxidants (Basel). 2026 Oct 16;15(10):1334. doi: 10.3390/antiox15101334 (PMC13701330; doi:10.3390/antiox15101334)
Supplement: Supplementary file 1 [file antioxidants-15-01334-s001.zip › antioxidants-4588915-supplementary.pdf]

Supplementary Materials

Erythrocyte Glutathione Shows a Larger Case–Control Difference than Serum Thiol-Disulfide Homeostasis at Emergency Department Presentation with Non-ST-Elevation Myocardial Infarction

Faruk Danış and Murat Alışık

Contents: Table S1, Table S2, Table S3, Table S4, Figure S1, Figure S2, Figure S3. All analyses were run on the 157 analyzed participants (82 patients with non-ST-elevation myocardial infarction, 75 controls). Values were produced by the same analysis scripts as the main text; abbreviations follow the main text.

Table S1. Covariate balance before and after 1:1 propensity-score matching on age, sex and current smoking (calliper 0.2 standard deviations of the logit propensity score).

Covariate	Before matching: NSTEMI (n = 82)	Before matching: controls (n = 75)	SMD before	After matching: NSTEMI (n = 38)	After matching: controls (n = 38)	SMD after
Age, years	59.2 (13.6)	53.3 (11.0)	0.47	54.3 (12.0)	56.3 (10.7)	−0.18
Male sex	65 (79.3%)	21 (28.0%)	1.20	23 (60.5%)	21 (55.3%)	0.11
Current smoking	44 (53.7%)	15 (20.0%)	0.74	12 (31.6%)	13 (34.2%)	−0.06
Hypertension	50 (61.0%)	0 (0.0%)	1.77	23 (60.5%)	0 (0.0%)	1.75
Diabetes mellitus	19 (23.2%)	0 (0.0%)	0.78	4 (10.5%)	0 (0.0%)	0.49
Hyperlipidaemia	34 (41.5%)	0 (0.0%)	1.19	14 (36.8%)	0 (0.0%)	1.08
Known coronary artery disease	23 (28.0%)	0 (0.0%)	0.88	10 (26.3%)	0 (0.0%)	0.85

Values are mean (standard deviation) for age and n (%) for binary covariates. SMD, standardized mean difference (difference in means or proportions divided by the pooled standard deviation; for the four comorbidities the control prevalence is zero, so the pooled standard deviation is taken from the patient group alone and the value is reported for completeness only). Propensity scores ranged from 0.15 to 0.96 in patients (median 0.76, IQR 0.54 to 0.90) and from 0.08 to 0.91 in controls (median 0.23, IQR 0.16 to 0.54). The region of common support was 0.15 to 0.91; 13 patients lay above it and 18 controls below it. Matching within a calliper of 0.31 logit units (0.2 × 1.55) produced 38 pairs; 44 patients and 37 controls were left unmatched and discarded from the matched analysis.

Table S2. Unadjusted and adjusted standardized case–control differences for all nine redox indices (linear models of each standardized index on case status, with and without age, sex and current smoking; n = 157).

Index	Type	Unadjusted β (95% CI)	Adjusted β (95% CI)	Adjusted p
Native thiol	Measured	−0.54 (−0.85 to −0.24)	−0.60 (−0.98 to −0.23)	0.002
Total thiol	Measured	−0.42 (−0.73 to −0.11)	−0.50 (−0.89 to −0.12)	0.011
Disulfide	Derived	0.49 (0.19 to 0.80)	0.40 (0.03 to 0.78)	0.037
Disulfide/native thiol	Derived	0.62 (0.32 to 0.92)	0.62 (0.25 to 0.99)	0.001
Native thiol/disulfide ratio	Derived	−0.19 (−0.50 to 0.13)	−0.33 (−0.71 to 0.06)	0.100
Reduced glutathione	Measured	−1.00 (−1.27 to −0.72)	−0.97 (−1.31 to −0.63)	<0.001
Total glutathione	Measured	−0.84 (−1.13 to −0.55)	−0.81 (−1.17 to −0.46)	<0.001
Oxidized glutathione	Derived	0.82 (0.53 to 1.11)	0.80 (0.44 to 1.15)	<0.001
GSSG/GSH	Derived	0.95 (0.68 to 1.23)	0.93 (0.58 to 1.27)	<0.001

β, difference between patients and controls in standard-deviation units of the index (negative values indicate lower values in patients); 95% confidence intervals from the model. Because the index is standardized on the whole sample, the unadjusted β is slightly smaller than Hedges g, which uses the pooled within-group standard deviation; the two scales rank the indices identically.

Table S2b. Adjusted odds of non-ST-elevation myocardial infarction per one standard deviation of the index (logistic regression with age, sex and current smoking; n = 157; Wald 95% confidence intervals).

Index	Odds ratio of NSTEMI per 1 SD (95% CI)	p
Reduced glutathione	0.30 (0.18 to 0.50)	<0.001
Native thiol	0.50 (0.31 to 0.79)	0.003
GSSG/GSH	3.51 (2.01 to 6.11)	<0.001
Disulfide/native thiol	1.90 (1.23 to 2.95)	0.004

Table S3. Spearman correlation matrix among the nine redox indices in all 157 participants.

Index	Native thiol	Total thiol	Disulfide	Disulfide/native thiol	Native thiol/disulfide ratio	Reduced glutathione	Total glutathione	Oxidized glutathione	GSSG/GSH
Native thiol	1.00	0.95	−0.15	−0.47	0.47	0.46	0.49	0.04	−0.18
Total thiol	0.95	1.00	0.11	−0.22	0.22	0.38	0.42	0.09	−0.11
Disulfide	−0.15	0.11	1.00	0.93	−0.93	−0.30	−0.28	0.18	0.29
Disulfide/native thiol	−0.47	−0.22	0.93	1.00	−1.00	−0.44	−0.43	0.16	0.34
Native thiol/disulfide ratio	0.47	0.22	−0.93	−1.00	1.00	0.44	0.43	−0.16	−0.34
Reduced glutathione	0.46	0.38	−0.30	−0.44	0.44	1.00	0.97	−0.22	−0.64
Total glutathione	0.49	0.42	−0.28	−0.43	0.43	0.97	1.00	−0.02	−0.47
Oxidized glutathione	0.04	0.09	0.18	0.16	−0.16	−0.22	−0.02	1.00	0.87
GSSG/GSH	−0.18	−0.11	0.29	0.34	−0.34	−0.64	−0.47	0.87	1.00

Native thiol, total thiol, reduced glutathione and total glutathione are measured directly; the remaining indices are calculated from them (disulfide = (total thiol − native thiol)/2; disulfide/native thiol = 100 × disulfide/native thiol; native thiol/disulfide ratio = native thiol/disulfide; oxidized glutathione = (total glutathione − reduced glutathione)/2; GSSG/GSH = 100 × oxidized/reduced glutathione). The two serum ratios are reciprocals, hence the correlation of −1.00. Matrices within patients and within controls show the same structure and are available from the corresponding author.

Table S4. Exploratory discrimination between patients and controls: area under the receiver operating characteristic curve (AUC) for each index, with DeLong 95% confidence intervals (n = 157).

Index	Type	AUC (95% CI)
Native thiol	Measured	0.65 (0.56 to 0.73)
Total thiol	Measured	0.62 (0.53 to 0.71)
Disulfide	Derived	0.63 (0.55 to 0.72)
Disulfide/native thiol	Derived	0.67 (0.59 to 0.76)
Native thiol/disulfide ratio	Derived	0.67 (0.59 to 0.76)
Reduced glutathione	Measured	0.80 (0.72 to 0.87)
Total glutathione	Measured	0.75 (0.67 to 0.83)
Oxidized glutathione	Derived	0.73 (0.65 to 0.81)
GSSG/GSH	Derived	0.81 (0.73 to 0.88)

Table S4b. Pairwise comparison of correlated AUCs by the DeLong method.

Comparison (first minus second)	AUC difference	z	p
Reduced glutathione vs Native thiol	0.148	3.29	0.001
GSSG/GSH vs Disulfide/native thiol	0.134	2.75	0.006
Reduced glutathione vs	0.123	2.69	0.007

Disulfide/native thiol			
GSSG/GSH vs Native thiol	0.158	2.97	0.003
Total glutathione vs Total thiol	0.133	2.77	0.006
Oxidized glutathione vs Disulfide	0.097	1.73	0.084
Reduced glutathione vs GSSG/GSH	−0.011	−0.31	0.758
Native thiol vs Disulfide/native thiol	−0.024	−0.51	0.611

These analyses were fitted and evaluated in the same sample without prespecified thresholds or external validation and support no diagnostic claim. For comparison, a classifier consisting only of the presence of any of hypertension, diabetes, hyperlipidaemia or known coronary artery disease, which were absent in controls by eligibility, gives an AUC of 0.93 (0.89 to 0.97) with sensitivity 0.85 and specificity 1.00, which illustrates that discrimination in this design reflects participant selection.

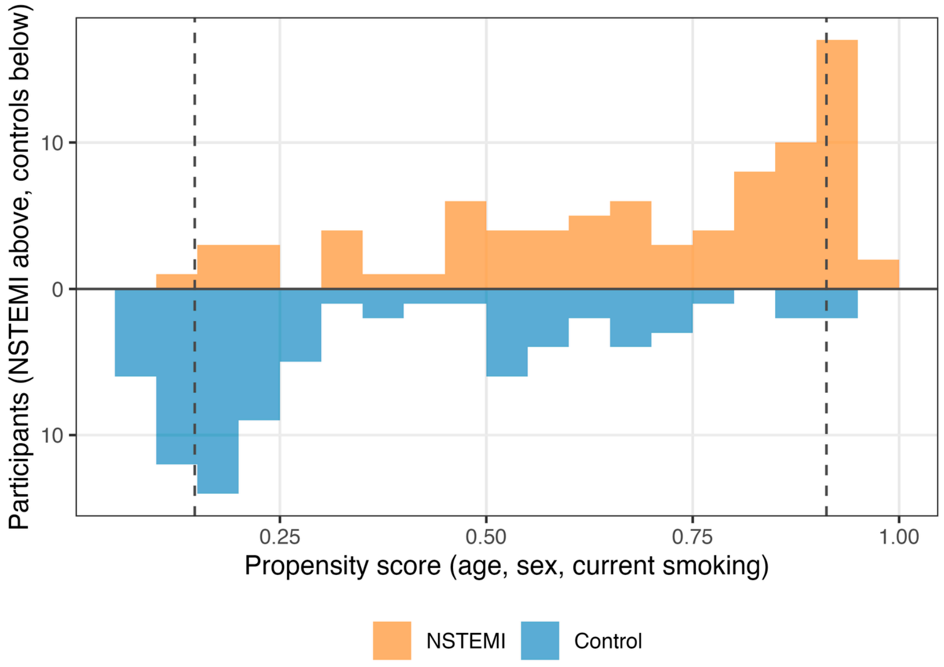

Figure S1. Propensity-score distributions (age, sex and current smoking) in patients with non-ST-elevation myocardial infarction (above the axis) and controls (below the axis). Dashed lines mark the region of common support.

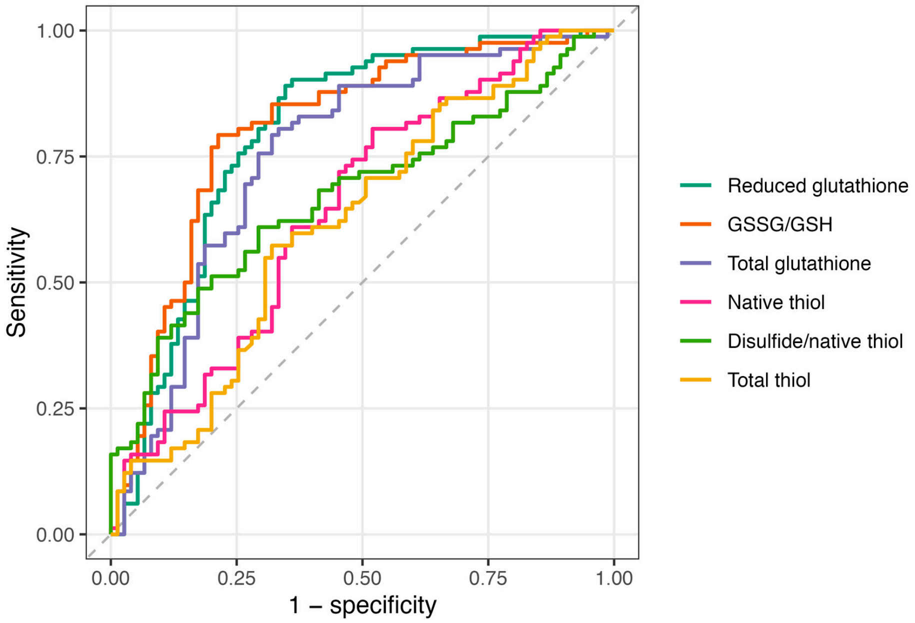

Figure S2. Receiver operating characteristic curves for the four directly measured indices and the two principal ratios (exploratory; fitted and evaluated in the same sample).

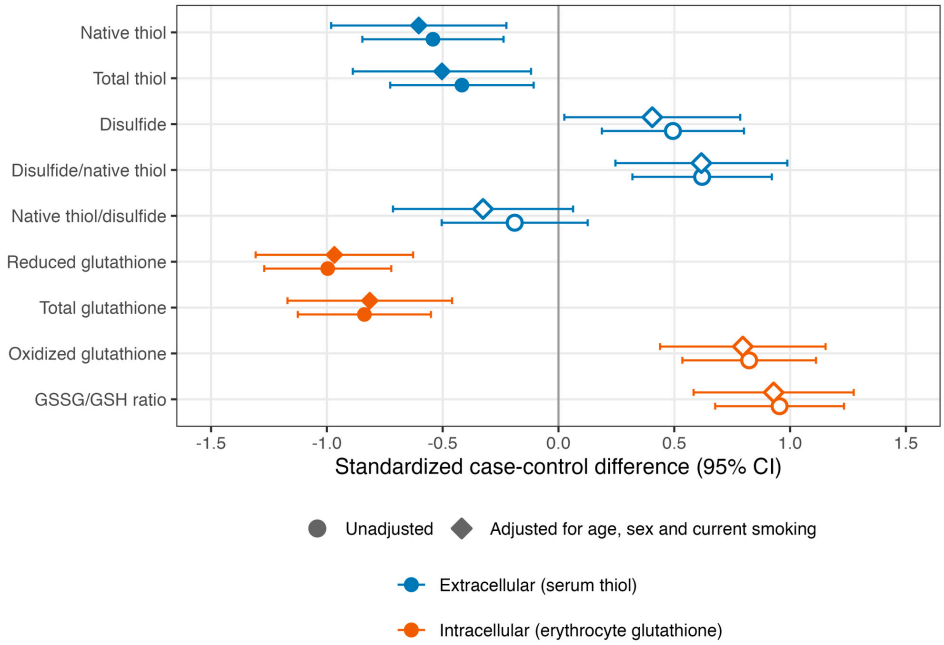

Figure S3. Unadjusted and adjusted standardized case–control differences for all nine redox indices on a common scale (β from linear models of each standardized index on case status, without and with age, sex and current smoking; n = 157; bars are 95% confidence intervals; negative values indicate lower values in patients). Filled symbols mark quantities measured directly and open symbols derived quantities. Values correspond to Table S2.
